# Mineral Nutrient Uptake, Accumulation, and Distribution in *Cunninghamia lanceolata* in Response to Drought Stress

**DOI:** 10.3390/plants12112140

**Published:** 2023-05-29

**Authors:** Shubin Li, Li Yang, Xiaoyan Huang, Zhiguang Zou, Maxiao Zhang, Wenjuan Guo, Shalom Daniel Addo-Danso, Lili Zhou

**Affiliations:** 1 Forestry College, Fujian Agriculture and Forestry University, Fuzhou 350002, China; fjlishubin@126.com (S.L.);; 2 Chinese Fir Engineering Technology Research Center of the State Forestry and Grassland Administration, Fuzhou 350002, China; 3 College of Resources and Environment, Fujian Agriculture and Forestry University, Fuzhou 350002, China; 4 Forest and Climate Change Division, CSIR-Forestry Research Institute of Ghana, Kumasi P.O. Box UP 63 KNUST, Ghana; sadanso@csir-forig.org.gh; 5 College of Geography and Oceanography, Minjiang University, Fuzhou 350108, China

**Keywords:** drought stress, *Cunninghamia lanceolata*, mineral elements, plant organs, nutrient uptake, nutrient distribution

## Abstract

Mineral accumulation in plants under drought stress is essential for drought tolerance. The distribution, survival, and growth of Chinese fir (*Cunninghamia lanceolata* (Lamb.) Hook.), an evergreen conifer, can be affected by climate change, particularly seasonal precipitation and drought. Hence, we designed a drought pot experiment, using 1-year-old Chinese fir plantlets, to evaluate drought effects under simulated mild drought, moderate drought, and severe drought, which corresponds to 60%, 50%, and 40% of soil field maximum moisture capacity, respectively. A treatment of 80% of soil field maximum moisture capacity was used as control. Effects of drought stress on mineral uptake, accumulation, and distribution in Chinese fir organs were determined under different drought stress regimes for 0–45 days. Severe drought stress significantly increased phosphorous (P) and potassium (K) uptake at 15, 30 and 45 days, respectively, within fine (diameter < 2 mm), moderate (diameter 2–5 mm), and large (diameter 5–10 mm) roots. Drought stress decreased magnesium (Mg) and manganese (Mn) uptake by fine roots and increased iron (Fe) uptake in fine and moderate roots but decreased Fe uptake in large roots. Severe drought stress increased P, K, calcium (Ca), Fe, sodium (Na), and aluminum (Al) accumulation in leaves after 45 days and increased Mg and Mn accumulation after 15 days. In stems, severe drought stress increased P, K, Ca, Fe, and Al in the phloem, and P, K, Mg, Na, and Al in the xylem. In branches, P, K, Ca, Fe, and Al concentrations increased in the phloem, and P, Mg, and Mn concentrations increased in the xylem under severe drought stress. Taken together, plants develop strategies to alleviate the adverse effects of drought stress, such as promoting the accumulation of P and K in most organs, regulating minerals concentration in the phloem and xylem, to prevent the occurrence of xylem embolism. The important roles of minerals in response to drought stress should be further evaluated.

## 1. Introduction

Mineral nutrients are irreplaceable in regulating plant metabolism and ecosystem functions [1]. Calcium (Ca) regulates soil pH and cation exchange capacity [2], iron (Fe) promotes nitrogen fixation in response to rising CO_2_ in tropical forests [3], and manganese (Mn) can enhance lignin degradation in the floors of oak and pine forests [4,5]. Drought stress is a vital abiotic factor affecting plant growth and survival. Moreover, drought stress may affect the uptake, accumulation, and distribution of mineral elements in plant organs because drought affects root growth and nutrient mobility in the soil and plant tissues [6,7]. A decrease in soil moisture reduced potassium (K) diffusion in the soil and root growth, which decreased K uptake by onion plants (*Allium cepa* L.) [8]. Drought stress can also reduce root and leaf phosphorous (P) concentrations in maize (*Zea mays*) and sorghum (*Sorghum bicolor* L.) [9,10]. However, the concentrations of magnesium (Mg), Ca, and K in sorghum tissues were not affected by drought stress [10].

Plant responses to drought stress include drought tolerance and avoidance. For instance, grain yield in wheat was negatively affected by drought stress, as a consequence of cellular oxidative damage due to elevation of malondialdehyde and hydrogen peroxide [11]. However, the presence of biomolecules, α-lipoic acid, and cysteine provided physiological tolerance and helped to restore yield attributes in wheat [11]. Moreover, seed primers such as *Bacillus thuringiensis* MH161336, silicon, and carrot extract boosted the morphological, physio-biochemical, and yield of pea plants under drought stress [12]. In addition, yeast extract and chitosan helped to reduce oxidative stress in garlic plants when exposed to drought, and subsequently resulted in improved productivity [13]. However, how the mineral uptake and accumulation in plants under stress affect drought tolerance is unclear. Soybeans grew better than rice (*Oryza sativa* L.) under drought stress because they maintained a higher nutrient uptake, especially K, which is a regulatory nutrient involved in osmotic adjustment [14]. Calcium, Mg, and P concentrations were found to be higher in the leaves of a drought-tolerant sorghum line (K866) than in those of a susceptible line (CS3541) [15]. Mineral elements have numerous functions in plants, such as maintaining charge balance, electron carriers, structural components, and enzyme activation and providing an osmoticum for plant growth [16]. Most studies on the effects of drought stress on mineral elements have focused on agriculture, especially crop yield, due in part to its relevance for food security [17,18]. However, studies on the role of mineral nutrients in alleviating drought stress in forests are scarce.

Chinese fir (*Cunninghamia lanceolata* (Lamb.) Hook), a fast-growing evergreen conifer, is an important timber species in subtropical China, covering an area of over 12 million hectares and accounting for approximately 6.5% of the world’s plantation areas [19]. Chinese fir has been cultivated for more than 3000 years and is distributed across 17 provinces, ranging from latitude 21°31′ to 34°03′ N and longitude 101°30′ to 121°53′ N [20]. The distribution, survival, and growth of Chinese fir can be affected by climate change, particularly seasonal precipitation and drought [21,22]. Zhang et al. [22] studied the effect of rainfall distribution on the soil water-holding capacity of Chinese fir plantations, and reported that mixed Chinese fir plantations showed a higher capacity to intercept and retain rainfall than monospecific plantations. It has also been demonstrated that nitrogen supply may enhance the antioxidant system (e.g., superoxide dismutase, peroxidase, and polyphenol oxidase) and nitrogen assimilation in Chinese fir under polyethylene glycol (PEG)-induced drought stress [21]. In addition, Sun et al. [23] used PEG-6000 to simulate drought stress and found that the chlorophyll fluorescence parameters varied substantially among different drought-tolerant Chinese fir clones. Previous studies on the effects of drought on Chinese fir have focused on physiological, hydraulic, and anatomical characteristics, drought tolerance evaluation, and screening [24,25,26]. To the best of our knowledge, no studies have investigated the effects of drought stress on the uptake, accumulation, and distribution of mineral elements in Chinese fir. Nonetheless, understanding these processes could help alleviate the adverse effects of drought stress and enable precise management and sustainable development of Chinese fir plantations.

We hypothesized that (1) mineral nutrient uptake by roots is greatly affected by drought stress, and the uptake effect varies among different root diameter classes, and (2) different drought stress intensities and durations affect mineral nutrient accumulation and distribution in different organs owing to different physiological requirements.

## 2. Results

### 2.1. Mineral Element Accumulation in C. lanceolata Plantlets under Drought Stress

Drought stress intensities, duration, and organs had significant effects (*p* < 0.001) on elemental concentrations (Table 1). The interactions between drought stress intensities × duration, identities × organs, durations × organs, and intensities × durations × organs were also significant (*p* < 0.001).

### 2.2. Effects of Drought Stress on the Mineral Element Uptake by Roots

**Figure 1:**
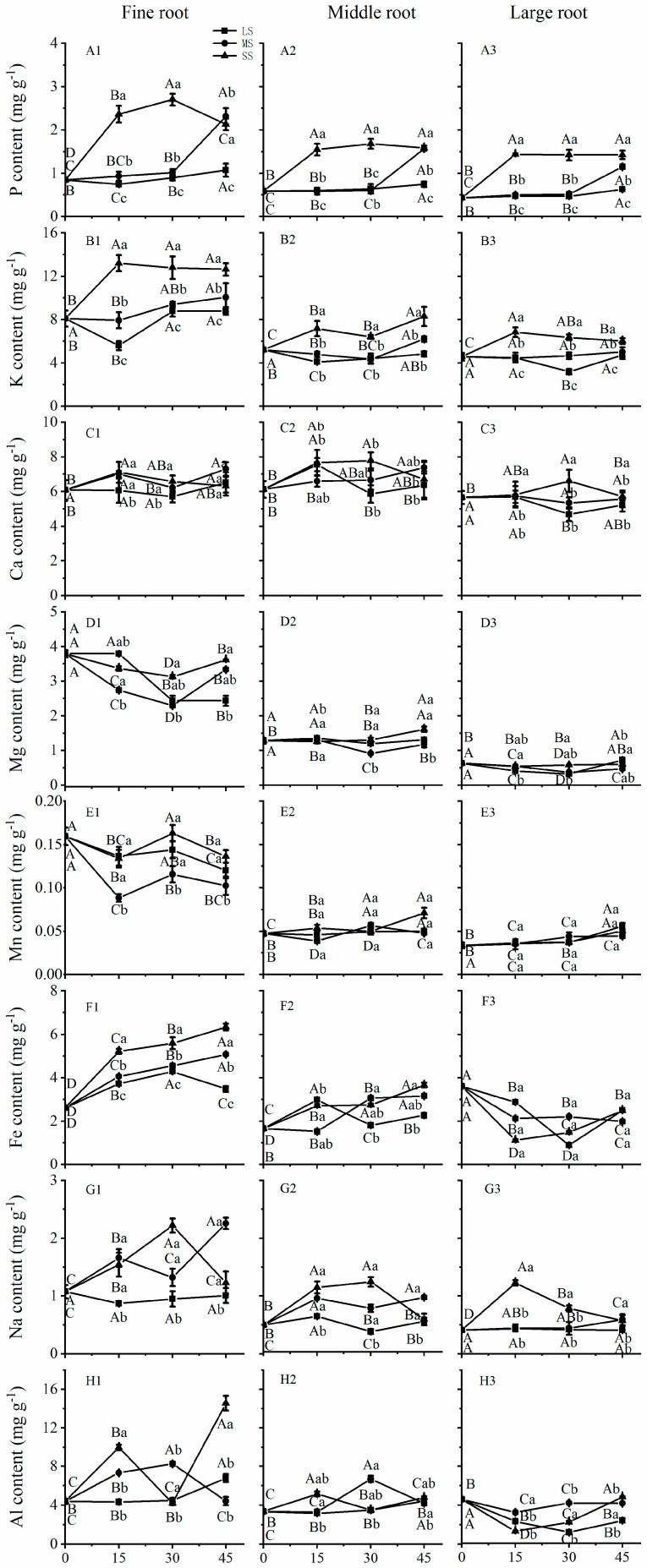
Uptake of mineral elements by fine, moderate, and large roots of *C. lanceolata* plantlets under different drought stress from 0 to 45 days. (**A1**–**A3**) represent P uptake by fine root, middle root and large root, respectively; (**B1**–**B3**) represent K uptake by fine root, middle root and large root, respectively; (**C1**–**C3**) represent Ca uptake by fine root, middle root and large root, respectively; (**D1**–**D3**) represent Mg uptake by fine root, middle root and large root, respectively; (**E1**–**E3**) represent Mn uptake by fine root, middle root and large root, respectively; (**F1**–**F3**) represent Fe uptake by fine root, middle root and large root, respectively; (**G1**–**G3**) represent Na uptake by fine root, middle root and large root, respectively; (**H1**–**H3**) represent Al uptake by fine root, middle root and large root, respectively. The squares, circles and triangles represent mild drought (LS), moderate drought (MS), and severe drought (SS), respectively. Different uppercase letters indicate significance among drought durations in the same drought stress intensity, and different lowercase letters indicate significance among drought stress intensities in the same drought duration. The differences were compared using ANOVA post-hoc means with Duncan’s analysis (*p* < 0.05). The values are presented as the mean ± standard deviation.

The effects of drought stress on elemental uptake by roots varied significantly among root diameters, indicating that the mean P, K, Mg, Mn, Na, Fe, and Al concentrations were higher in fine roots than in moderate and large roots (*p* < 0.01) (Figure 1). Among different diameter roots, compared to CK, severe drought stress significantly increased the mean P and K uptake by 180.96% and 50.74%, respectively, at Day 15, and the concentrations were maintained at high levels with increased drought time (Figure 1A,B). At 30 days, severe drought stress increased P uptake by 218.37%, 184.42%, and 228.86% in fine, moderate, and large roots, respectively (Figure 1(A1–A3)), and increased K uptake by 59.08%, 40.04%, and 40.30% in these roots, respectively, over 45 days (Figure 1(B1–B3)).

Ca and Mn concentrations in large roots did not change after 15 days of drought stress, indicating that Ca and Mn uptake was not sensitive to drought stress in large roots during earlier drought periods (Figure 1(C3,E3)), whereas fine roots showed decreased Mn uptake at Day 15 (Figure 1(E1)). Decreased Mg uptake was observed in fine roots at Days 15 and 30 but increased at Day 45. However, no effect on Mg uptake was observed during 45 days in moderate and large roots (Figure 1(D1–D3)). The Fe concentration increased in fine and moderate roots but decreased in large roots under drought stress (Figure 1(F1–F3)). Severe drought stress increased Na uptake at 15 and 30 days in fine and moderate roots, then decreased to the level of the control at Day 45 (Figure 1(G1–G3)). Al uptake increased at 15 days, decreased at 30 days, and then increased again at 45 days in fine and moderate roots, whereas Al uptake decreased at 15 days, and then gently increased at 30 and 45 days in large roots (Figure 1(H1–H3)).

### 2.3. Effects of Drought Stress on Mineral Elements Accumulations in Stems

**Figure 2:**
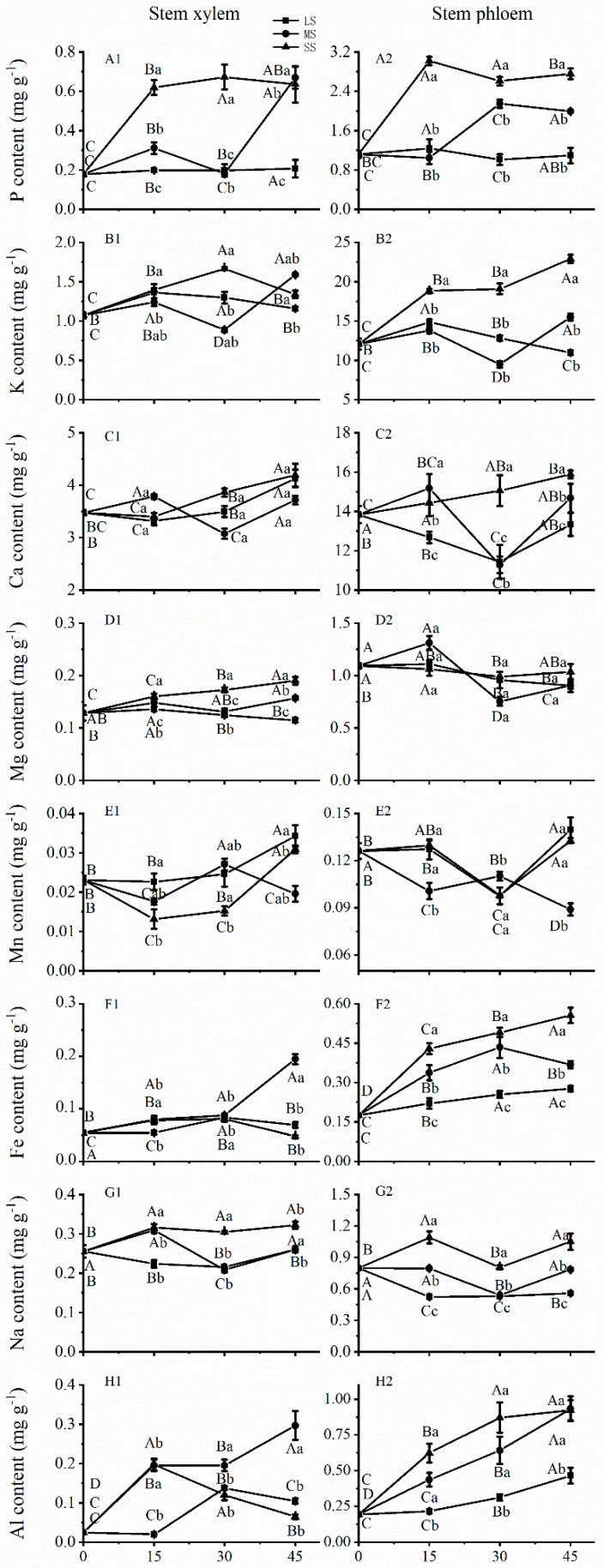
Accumulation of mineral elements in the xylem and phloem of stems of *C. lanceolata* plantlets under different drought stress from 0 to 45 days. (**A1**,**A2**) represent P accumulation in stem xylem and stem phloem, respectively; (**B1**,**B2**) represent K accumulation in stem xylem and stem phloem, respectively; (**C1**,**C2**) represent Ca accumulation in stem xylem and stem phloem, respectively; (**D1**,**D2**) represent Mg accumulation in stem xylem and stem phloem, respectively; (**E1**,**E2**) represent Mn accumulation in stem xylem and stem phloem, respectively; (**F1**,**F2**) represent Fe accumulation in stem xylem and stem phloem, respectively; (**G1**,**G2**) represent Na accumulation in stem xylem and stem phloem, respectively; (**H1**,**H2**) represent Al accumulation in stem xylem and stem phloem, respectively. The squares, circles and triangles represent mild drought (LS), moderate drought (MS), and severe drought (SS), respectively. Different uppercase letters indicate significance among drought durations in the same drought stress intensity, and different lowercase letters indicate significance among drought stress intensities in the same drought duration. The differences were compared using ANOVA post-hoc means with Duncan’s analysis (*p* < 0.05). The values are presented as the mean ± standard deviation.

The mean mineral concentration was significantly higher in the phloem than in the xylem (Figure 2). For the stem phloem, severe drought stress considerably increased P, K, Fe, and Al accumulation at 15 days, but the increase was gradual in the later drought period. Under severe drought stress for 45 days, their mineral concentrations increased by 146.87%, 89.95%, 219.91%, and 377.79%, respectively (Figure 2(A2,B2,F2,H2)). For the stem xylem, the effects of drought stress on mineral elements were similar to those on the stem phloem. Severe drought stress also substantially increased P, K, Mg, Na, and Al concentrations at 15 days, with a higher concentration level maintained in the xylem with increasing drought stress (Figure 2(A1,B1,D1,G1,H1)). 

### 2.4. Effects of Drought Stress on Mineral Element Accumulation in the Primary Lateral Branch

**Figure 3:**
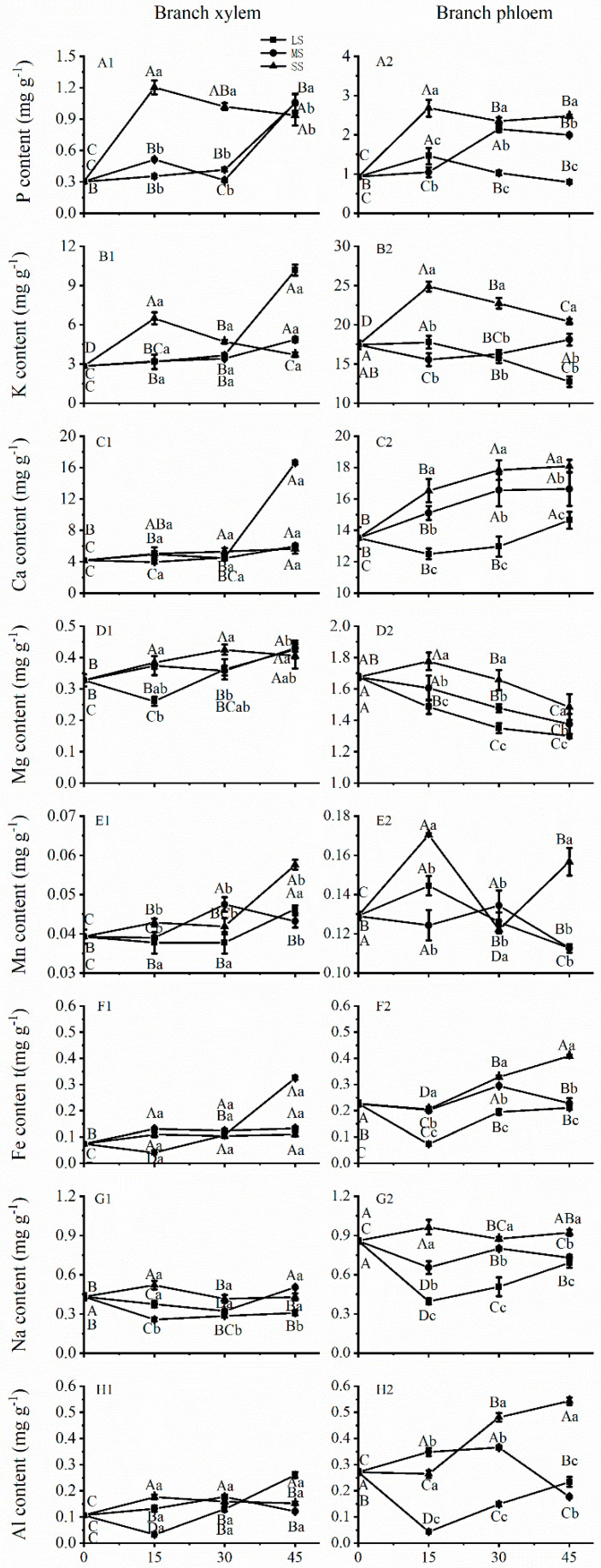
Accumulation of mineral elements in the xylem and phloem in branches of *C. lanceolata* plantlets under different drought stress from 0 to 45 days. (**A1**,**A2**) represent P accumulation in branch xylem and branch phloem, respectively; (**B1**,**B2**) represent K accumulation in branch xylem and branch phloem, respectively; (**C1**,**C2**) represent Ca accumulation in branch xylem and branch phloem, respectively; (**D1**,**D2**) represent Mg accumulation in branch xylem and branch phloem, respectively; (**E1**,**E2**) represent Mn accumulation in branch xylem and branch phloem, respectively; (**F1**,**F2**) represent Fe accumulation in branch xylem and branch phloem, respectively; (**G1**,**G2**) represent Na accumulation in branch xylem and branch phloem, respectively; (**H1**,**H2**) represent Al accumulation in branch xylem and branch phloem, respectively. The squares, circles and triangles represent mild drought (LS), moderate drought (MS), and severe drought (SS), respectively. Different uppercase letters indicate significance among drought durations in the same drought stress intensity, and different lowercase letters indicate significance among drought stress intensities in the same drought duration. The differences were compared using ANOVA post-hoc means with Duncan’s analysis (*p* < 0.05). The values are presented as the mean ± standard deviation.

Compared to the effects of mild and moderate stress on P, K, and Ca levels, severe drought stress significantly increased P, K, and Ca levels by 187.99%, 42.69%, and 22.43%, respectively, at Day 15, and then different increasing trends with increasing drought stress were maintained in the phloem (Figure 3(A2,B2,C2)). Fe and Al concentrations in the phloem were not sensitive to drought stress at 15 days, but with increasing stress, Fe and Al accumulation significantly increased at 30 and 45 days (Figure 3(F2,H2)). Drought stress significantly decreased Mg concentrations in the phloem from 15 to 45 days (Figure 3(D2)). Moreover, P and K concentrations in the xylem significantly increased during severe drought stress at 15 days, and higher mineral levels were maintained until 45 days (Figure 3(A1,B1)). In contrast, severe drought stress increased Mg accumulation at 15 days and reached the maximum value at 30 days in the xylem (Figure 3(D1)). The response of Mn concentration to severe drought stress was not sensitive at 15 and 30 days but significantly increased in the xylem at 45 days in the xylem (Figure 3(E1)).

### 2.5. Effects of Drought Stress on Mineral Element Accumulation in Leaves

**Figure 4:**
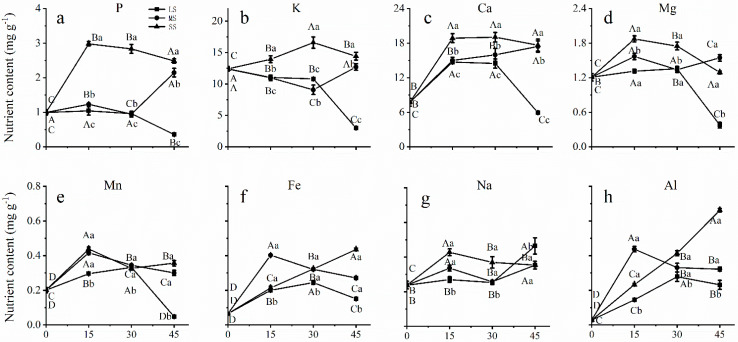
Mineral element accumulation in leaves of *C. lanceolata* plantlets under different drought stress from 0 to 45 days. The squares, circles and triangles represent mild drought (LS), moderate drought (MS), and severe drought (SS), respectively. Different uppercase letters indicate significance among drought durations in the same drought stress intensity, and different lowercase letters indicate significance among drought stress intensities in the same drought duration. The differences were compared using ANOVA post-hoc means with Duncan’s analysis (*p* < 0.05). The values are presented as the mean ± standard deviation.

In leaves, severe drought significantly increased P and Ca concentrations at 15 days, and then the concentrations decreased slightly, but still remained at a high level during the late stage of drought stress (Figure 4a,c). Severe drought stress slowly increased K accumulation to a maximum level at Day 30, followed by a decrease to the level of the control (Figure 4b). Mg and Mn concentrations significantly increased under severe drought stress in leaves at 15 days but subsequently decreased to the level of the control at 45 days (Figure 4d,e). With increasing drought stress, Fe and Al concentrations in the leaves increased sharply increased by 575.17% and 2242.04%, respectively, at 45 days (Figure 4f,g). Under drought stress for 15 days, Na concentrations increased among different drought intensities and decreased slightly at Day 30. However, the Na concentration was higher under drought stress conditions than under control conditions at 45 days (Figure 4h).

## 3. Discussion

### 3.1. Effect of Drought Stress on Mineral Element Uptake by Roots

The results are consistent with our first hypothesis that drought stress would affect mineral element uptake by the roots. Different drought stress intensities substantially increased P and K uptake by fine, moderate, and large roots at 15 days and maintained higher concentrations in the late drought stage. This result is inconsistent with those of other studies that reported a decline in the root uptake of P and K owing to the restricted transpiration rate and reduced diffusion in the soil [27,28,29]. Moreover, drought stress increases P and K concentrations in the roots of Pistacia cultivars [30]. K is essential in minimizing the adverse effects of drought stress on plant survival and crop production. The enhanced internal need for K by plants is due to increased reactive oxygen species (ROS) production and maintenance of photosynthetic CO_2_ fixation [31,32].

Mg and Mn uptake by fine roots showed a similar response trend, which decreased during the early stage of the different-intensity droughts and then increased in the late stage, indicating promotion of the uptake of these two minerals, whereas the increase in Ca uptake in the early drought stage prevented Mg and Mn uptake by fine roots. The uptake of certain minerals is promoted or inhibited by other elements, and at very high levels, Ca and Al in acidic soils reportedly compete with Mg for uptake, which decreases Mg uptake by roots [32,33].

Mineral uptake varied among different diameter roots. Mineral uptake was generally higher in fine roots compared to the other roots under drought stress (Figure 1). With increasing drought stress, Fe uptake increased in fine and moderate roots, with a significant increase in fine roots, but decreased in large roots (Figure 1(F1)). The higher nutrient availability in fine roots and a lower nutrient uptake and assimilation ability of large roots are consistent with the results of a previous study [34]. The higher nutrient availability in fine roots is because roots with different diameters have different water and nutrient uptake, anchoring, and supporting functions [35].

Commonly, absorptive fine roots have a higher specific root length (SRL) and greater water and nutrient uptake rates than roots with larger diameters, which often have lower SRL [36,37]. However, roots use multiple mineral uptake strategies. Roots may enhance SRL and increase root number, biomass, and carboxylic acid exudation to improve nutrient availability [38]. In southern China, soils are acidic, available P is low, and P is fixed as Fe and Al. In this study, increased P uptake under drought stress may be due to the organic acid exudates from roots that mobilize soluble Al-P and Fe-P [39]. Simultaneously, soluble Fe^2+^and Al^3+^ were increased by exudate dissolution in the soil and promoted the uptake of these two minerals by fine and moderate roots. 

### 3.2. Effect of Drought Stress on Mineral Element Accumulation and Distribution among Different Organs

Drought stress affected the accumulation and distribution of different minerals among different organs, which is an essential trade-off strategy to mitigate the adverse effects of drought stress. Our results also supported the second hypothesis that different drought stresses could affect mineral nutrient accumulation and distribution in different organs. In this study, severe drought stress enhanced the P and K accumulation in all organs except for K in the branch xylem. Drought stress decreased Mg and Mn accumulation in fine roots and increased the concentration in leaves at 15 days. Na and Al can be toxic to plants, significantly accumulated in leaves under drought stress, and may have influenced the physiological equilibrium and photosynthetic activity in the leaves.

Drought stress markedly increased Ca distribution in leaves compared to that in other organs. The roots, stems, branches, and leaves play distinct roles in ecosystem functions. Mineral elements are commonly allocated to resource-acquiring organs (i.e., leaves and fine roots) rather than other tissues, and the nutrient concentration in the roots and leaves is relatively higher [40]. Most biomass in stems and branches is also essential for internal nutrient recycling, but nutrient concentrations within stems remain poorly understood compared to those in leaves and roots [41].

P, K, Ca, Fe, and Al concentrations in the phloem of stems and branches increased under severe drought stress compared to those in the control, indicating that these minerals accumulated in the stems and branches. The P and K accumulation in these organs can balance osmotic adjustments, prevent embolisms, and mitigate the adverse effects of drought stress. However, the role of minerals in the stem in response to drought stress has rarely been evaluated. The stems are the primary transport media for water and nutrients; the phloem is a major nutrient transport organ, and the xylem plays supporting and transporting roles [42]. Water deficits restrict water flow and vertical translocation, leading to hydraulic failure and asymmetrical changes in nutrient partitioning between different organs [43]. Mineral concentration variations in the phloem and xylem under drought stress may be vital in osmotic adjustment, which regulates water and mineral transport and alleviates the adverse effects of drought stress.

### 3.3. Function of Mineral Nutrients in Drought Stress Alleviation

Drought stress adversely influences normal physiology and plant growth. Water stress inhibits photosynthesis in plants by closing stomata and damaging the photosynthetic apparatus, causing considerable ROS accumulation and inducing oxidative stress damage of proteins, membrane lipids, and other cellular components [6]. Mineral elements are vital for maintaining charge balance, electron carriers, enzyme activation, and osmoticum for turgor and growth [16].

An increase in xylem K^+^ can lead to enhanced xylem hydraulic conductivity, which substantially alleviated embolism-induced water reduction during vertical water transportation [44]. These results are consistent with the findings of this study, which showed increased K concentrations in the xylem and phloem of stems under severe and moderate drought stress at 15 and 30 days (Figure 2(B1,B2)), thus helping to alleviate stem embolism. However, the K concentration in the branch xylem showed a decreased trend under different drought stress conditions at Day 30 and Day 45, which may increase the possibility of embolism in the branches (Figure 3(B1,B2)). K plays a role in photosynthesis, enzyme activation, and osmotic potential. Moreover, K^+^ was found to be the most prevalent mineral in the xylem and phloem of *Ricinus communis* plants, and its phloem motility is considered high [42]. Drought stress increased K, Ca, and Na contents and decreased Fe and Mg contents in leaves; the concentrations varied among castor plant ecotypes [45].

Ca plays a role in plant structures (membranes and cell walls), and free Ca^2+^ acts as a critical link between environmental signals and certain physiological responses [46]. In the present study, Ca accumulation and distribution in the leaves increased under severe and moderate drought stress, consistent with the findings of Peuke [42], who found that Ca increased to 79% in the shoots. Generally, Ca^2+^ activity is higher in sieve tubes and adjacent cells and is driven by hydrostatic pressure differences regulated by the “demand of the shoot” [28,47]. However, the mobility of intra- and intercellular Ca^2+^ is relatively low during long-distance transport [48].

Acidic soil is characterized by a rapid increase in Al concentration with a decrease in soil moisture, in contrast to limed soil, where only slight changes in Al concentration could occur with drought stress [49]. Drought stress resulted in Al uptake through the root surface and substantial accumulation in leaves (Figure 1H and Figure 4h). Al accumulation in leaves under severe drought stress may negatively affect tree growth and root system development [50]. In our study, the increased accumulation of Na and Al in the leaves of Chinese fir may be negatively affected by drought stress, but the increased uptake and accumulation of P and K by the roots, stems, and leaves could mitigate the adverse effects of drought. Therefore, the changing concentrations of minerals in Chinese fir under drought stress in this study and the results of other studies [51] suggest that nutrient uptake and allocation may play an essential role in the response of plants to drought stress, and this points to need for further studies to evaluate the regulatory mechanisms of different minerals in plant organs. To address the limitations of this study, future research should determine the important role of minerals in plant responses to drought stress, and the relationship between mineral uptake, allocation, and xylem embolism should also be elucidated.

## 4. Materials and Methods

### 4.1. Plant Materials

The experiment was conducted in the Intensive Breeding Greenhouse of the Chinese Fir Engineering Technology Research Center, National Forestry and Grass Bureau of Fujian Agriculture and Forestry University. Original plant materials were obtained from a third-generation Chinese fir seed orchard on Youxi National Farm, Fujian Province. In early July 2021, approximately 100 saplings of *C. lanceolata* were planted in pots. The pots were approximately 30 cm in diameter and 35 cm in height. After 3 months of cultivation and domestication with an adequate water supply to keep soil in the maximum field capacity (44.83%), we started the drought stress experiment in mid-November 2021. We selected 40 healthy, straight, and well-growing seedlings as study materials. The seedlings were approximately 40 cm in height and 0.50 cm in basal diameter. The seedlings were subjected to the following drought treatments: 80% of the maximum field capacity (CK), mild drought stress, 60% of the maximum field capacity (LS), moderate drought stress, 50% of the maximum field capacity (MS), and severe drought stress, 40% of the maximum field capacity (SS). We irrigated all the treatments every 2–3 days to maintain the corresponding water level. A soil moisture monitor (TZS-2X, Zhejiang Top Cloud-Agri Technology Co., Ltd., Hangzhou, China) was used to monitor the soil moisture and maintain the soil water content within the prescribed range. Samples were taken on Days 15, 30, and 45 after the drought stress treatment, and each sampling comprised four replicates.

### 4.2. Samples and Mineral Measurements

The seedlings were divided into roots, stems, branches, and leaves at each sampling point. Roots were further divided into different diameter classes: fine roots (diameter < 2 mm), moderate roots (diameter 2–5 mm), and large roots (diameter 5–10 mm). The phloem and xylem in stems and primary branches were sampled as previously described [52]. All the samples were placed in an oven at 105 °C for 15 min to deactivate enzymes and then at 80 °C for at least 48 h to dry to a constant weight [20]. For each sample, a 0.1 g subsample was digested using HNO_3_-H_2_SO_4_ solution (volume ratio 5:1). Total P was measured using the molybdenum-antimony colorimetric method with a spectrophotometer (AOXI Instruments, Shanghai MADAPA Instruments Co., Ltd., Shanghai, China). The absorbance of the solution was determined at 700 nm using ultrapure water as the blank. The total potassium (K), calcium (Ca), magnesium (Mg), manganese (Mn), iron (Fe), sodium (Na), and aluminum (Al) contents were determined using an Inductively Coupled Plasma Optical Emission Spectroscopy (ICP-OES, OPTIMA 8000, Perkin Elmer, Waltham, MA, USA).

### 4.3. Statistical Analysis

The data are presented as the means ± SE. The effects of drought stress, duration, and organ and element concentration interactions were tested using a three-way analysis of variance (ANOVA). The fixed factors were stress type, stress duration, and organs. ANOVA was used to compare element concentrations among different stress intensities or durations. The means were compared using Duncan’s post-hoc test (*p* < 0.05). All statistical analyses were performed using SPSS 22.0 Software package for Windows (SPSS Inc., Chicago, IL, USA)

## 5. Conclusions

Minerals not only play a vital role in maintaining plant growth, but also are important in alleviating the negative effects of drought. Effects of drought stress on different mineral uptake varied among root diameters. Severe drought increased P and K uptake through all diameter roots, while Mg, Mn, and Fe uptake was enhanced by fine roots under severe drought. For branches and stems, the P, K, Ca, Fe, and Al concentrations were increased in the phloem, and P and Mg was increased in the xylem under severe drought stress. Severe drought increased P, K, Ca, Fe, Na, and Al accumulation in leaves after 45 days. We can conclude that the plant increased P and K uptake by different diameter roots, and accumulated in all organs, in order to support plant survival and growth under drought conditions. The increased mineral concentrations in the phloem and xylem gave us new cues to study how to prevent embolism in the xylem water transport network for plants.

## Figures and Tables

**Table 1:** Three-way ANOVA analysis of drought stress intensities (degree of freedom, df = 2), stress durations (df = 3), organs (df = 7), and their interaction on mineral elements of *C. lanceolata* plantlets under drought stress.

Values	P	K	Ca	Mg	Mn	Fe	Na	Al
Stress idensities	3502.748 ***	1467.197 ***	183.548 ***	407.141 ***	113.537 ***	582.831 ***	728.439 ***	682.979 ***
Stress durations	212.635 ***	21.721 ***	38.051 ***	247.635 ***	24.621 ***	332.982 ***	15.615 ***	303.519 ***
Organs	978.657 ***	3802.481 ***	2165.212 ***	11,501.378 ***	5371.569 ***	19,024.356 ***	1048.194 ***	7037.542 ***
Idensities × Durations	318.770 ***	67.458 ***	15.713 ***	105.564 ***	101.988 ***	224.430 ***	103.971 ***	437.225 ***
Idensities × Organs	94.763 ***	129.886 ***	83.617 ***	82.185 ***	440.899 ***	333.135 ***	67.189 ***	261.831 ***
Durations × Organs	17.369 ***	43.222 ***	54.137 ***	113.068 ***	437.870 ***	99.668 ***	15.673 ***	122.913 ***
Idensities × Durations × Organs	332.877 ***	54.211 ***	44.892 ***	74.579 ***	278.240 ***	140.507 ***	33.498 ***	231.321 ***

Note: The F values are presented in the table. *** indicate significant differences at *p* < 0.001.

## Data Availability

All analysed data are presented in the main text.
